# Cell Type-Specific mRNA Dysregulation in Hippocampal CA1 Pyramidal Neurons of the Fragile X Syndrome Mouse Model

**DOI:** 10.3389/fnmol.2017.00340

**Published:** 2017-10-20

**Authors:** Laura Ceolin, Nathalie Bouquier, Jihane Vitre-Boubaker, Stéphanie Rialle, Dany Severac, Emmanuel Valjent, Julie Perroy, Emma Puighermanal

**Affiliations:** ^1^IGF, CNRS, INSERM, Univ. Montpellier, Montpellier, France; ^2^Montpellier GenomiX c/o IGF, Montpellier, France

**Keywords:** FXS, functional genomics, KLK8, RiboTag, spine maturation

## Abstract

Fragile X syndrome (FXS) is a genetic disorder due to the silencing of the *Fmr1* gene, causing intellectual disability, seizures, hyperactivity, and social anxiety. All these symptoms result from the loss of expression of the RNA binding protein fragile X mental retardation protein (FMRP), which alters the neurodevelopmental program to abnormal wiring of specific circuits. Aberrant mRNAs translation associated with the loss of *Fmr1* product is widely suspected to be in part the cause of FXS. However, precise gene expression changes involved in this disorder have yet to be defined. The objective of this study was to identify the set of mistranslated mRNAs that could contribute to neurological deficits in FXS. We used the RiboTag approach and RNA sequencing to provide an exhaustive listing of genes whose mRNAs are differentially translated in hippocampal CA1 pyramidal neurons as the integrative result of FMRP loss and subsequent neurodevelopmental adaptations. Among genes differentially regulated between adult WT and *Fmr1*^−/*y*^ mice, we found enrichment in FMRP-binders but also a majority of non-FMRP-binders. Interestingly, both up- and down-regulation of specific gene expression is relevant to fully understand the molecular deficiencies triggering FXS. More importantly, functional genomic analysis highlighted the importance of genes involved in neuronal connectivity. Among them, we show that *Klk8* altered expression participates in the abnormal hippocampal dendritic spine maturation observed in a mouse model of FXS.

## Introduction

Fragile X syndrome (FXS) is an inherited neurodevelopmental disorder with a wide variety of symptoms, including intellectual disability, seizures, hyperactivity, social anxiety, and others characteristic of autism spectrum disorders. It is caused by loss-of-function mutations in the RNA binding protein fragile X mental retardation protein (FMRP) (Pieretti et al., 1991; Kelleher and Bear, 2008; Lozano et al., 2014). The knockout of *Fmr1* gene in mouse (*Fmr1*^−/*y*^ mice) exhibits the primary molecular and behavioral symptoms associated with FXS (Hou et al., 2006). Many pathological changes observed in FXS are thought to be a result of a modest increase in protein synthesis (Bear et al., 2004; Bhakar et al., 2012; Bhattacharya et al., 2012). Indeed, previous studies have shown a rescue of altered synaptic plasticity and some neurological symptoms by normalizing the rate of global mRNA translation (Dolen et al., 2007; Osterweil et al., 2010, 2013; Krueger and Bear, 2011; Qin et al., 2015). However, the mRNAs that are aberrantly translated remain to be identified.

In the search for genes expression deficiencies, quantitative genomic approaches are complicated by complex RNA profiles from individual cell types within a tissue. Genetically labeled cell types strategies now allow focusing on a pertinent cell type to identify specific gene expression (Heiman et al., 2008; Sanz et al., 2009). We chose the RiboTag approach (Sanz et al., 2009), which is a methodology for affinity purification of ribosome-bound mRNAs from genetically defined cell populations in the brain. The RiboTag mouse line expresses the ribosomal protein Rpl22 tagged with the hemagglutinin (HA) epitope in specific cell types by mating to a Cre recombinase-expressing mouse. HA-tagged ribosomes can be then purified from the target cell population and their associated mRNAs sequenced. This allows the comparison of translatome profiles in a genetically-identified cell population between mouse genotypes.

FMRP loss in *Fmr1*^−/*y*^ mice has been shown to cause abnormal synaptic and structural plasticity in CA1 pyramidal cells (Huber et al., 2002; Lauterborn et al., 2007; Hu et al., 2008; Meredith and Mansvelder, 2010; Busquets-Garcia et al., 2013), which in turn have been associated with impaired hippocampal function as well as cognitive deficits (Contractor et al., 2015; Radwan et al., 2016). We thus studied the mRNA translation in hippocampal CA1-pyramidal cells in *Fmr1*^−/*y*^ mice compared to wild-type littermates in order to identify those genes involved in this neurodevelopmental disorder. Differential analysis of ribosome-associated mRNA revealed up- and down-regulation of genes linked to plasticity-related functions. Among them, we found a decreased expression of *Klk8* in *Fmr1*^−/*y*^ mice. KLK8, Kallikrein Related Peptidase 8 (also known as neuropsin), is a serine protease expressed focally in the limbic system (Chen et al., 1995), especially in hippocampal CA1 pyramidal cells, which drives early processes of memory acquisition and Schaffer collateral plasticity in adult mouse hippocampus (Tamura et al., 2006). Interestingly, KLK8 catalyzes the proteolysis of proteins from the extracellular matrix (Matsumoto-Miyai et al., 2003) and could thus control adhesion changes between pre- and postsynaptic neurons needed for stable synaptic plasticity. Here, we show that re-establishing KLK8 expression in *Fmr1*^−/*y*^ cultured hippocampal neurons restores normal dendritic spine maturation.

## Results

**Figure 1:**
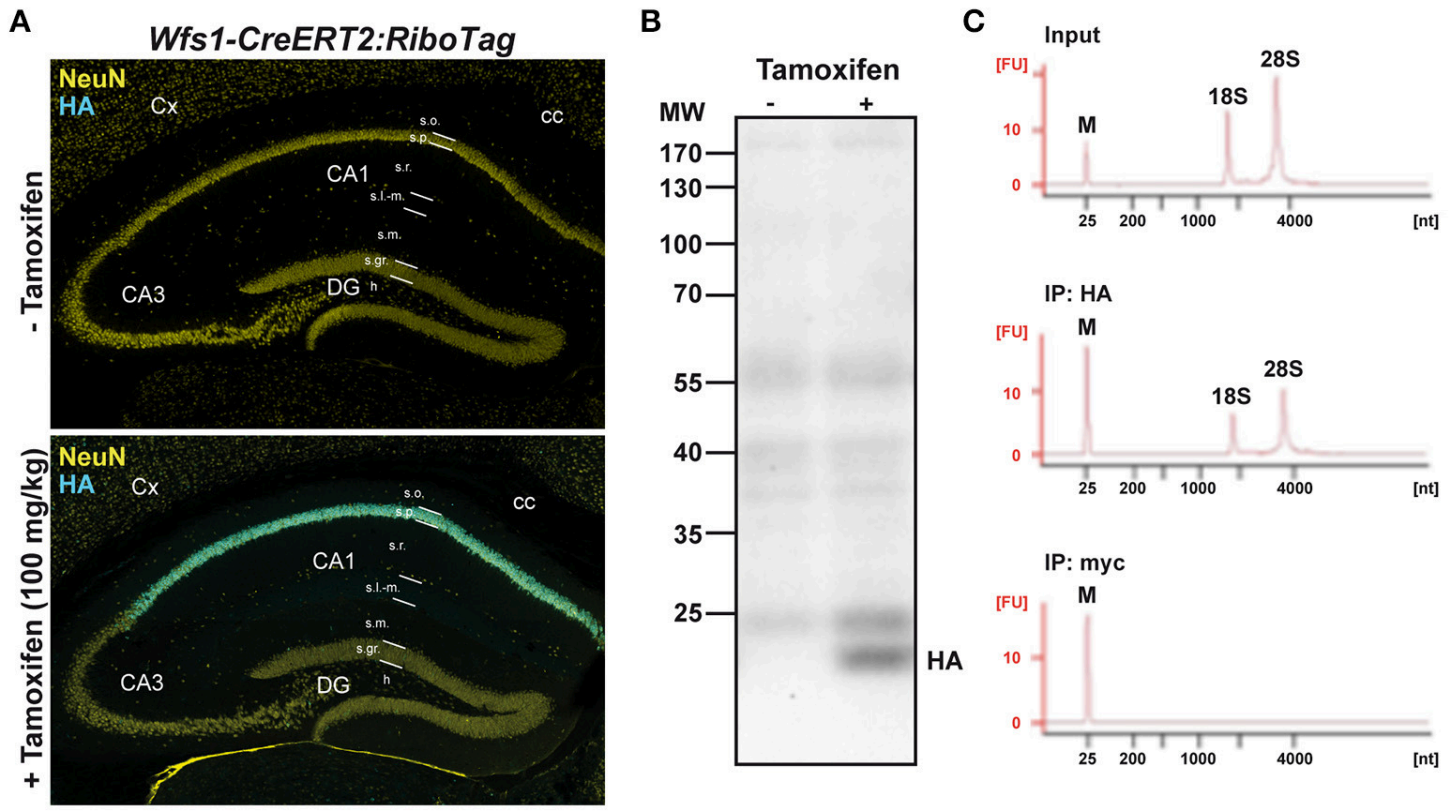
Characterization of *Wfs1-CreERT2:RiboTag* mouse line. **(A)** Double immunofluorescence HA (cyan) and NeuN (yellow) in *Wfs1-CreERT2:RiboTag* mice treated with vehicle (top) or tamoxifen (bottom, 100 mg/kg) for 3 consecutive days and sacrificed 1 week later. **(B)** Immunoblot of HA of hippocampal homogenates from *Wfs1-CreERT2:RiboTag* mice treated with vehicle or tamoxifen (100 mg/kg) for 3 consecutive days and sacrificed 1 week later. **(C)** Specificity of HA-tagged ribosomes immunoprecipitation. Agilent Technologies 2100 Bioanalyzer electropherogram analysis of hippocampal homogenate (input) and immunoprecipitates using anti-HA and control anti-Myc antibodies in *Wfs1-CreERT2:RiboTag* mice.

**Figure 2:**
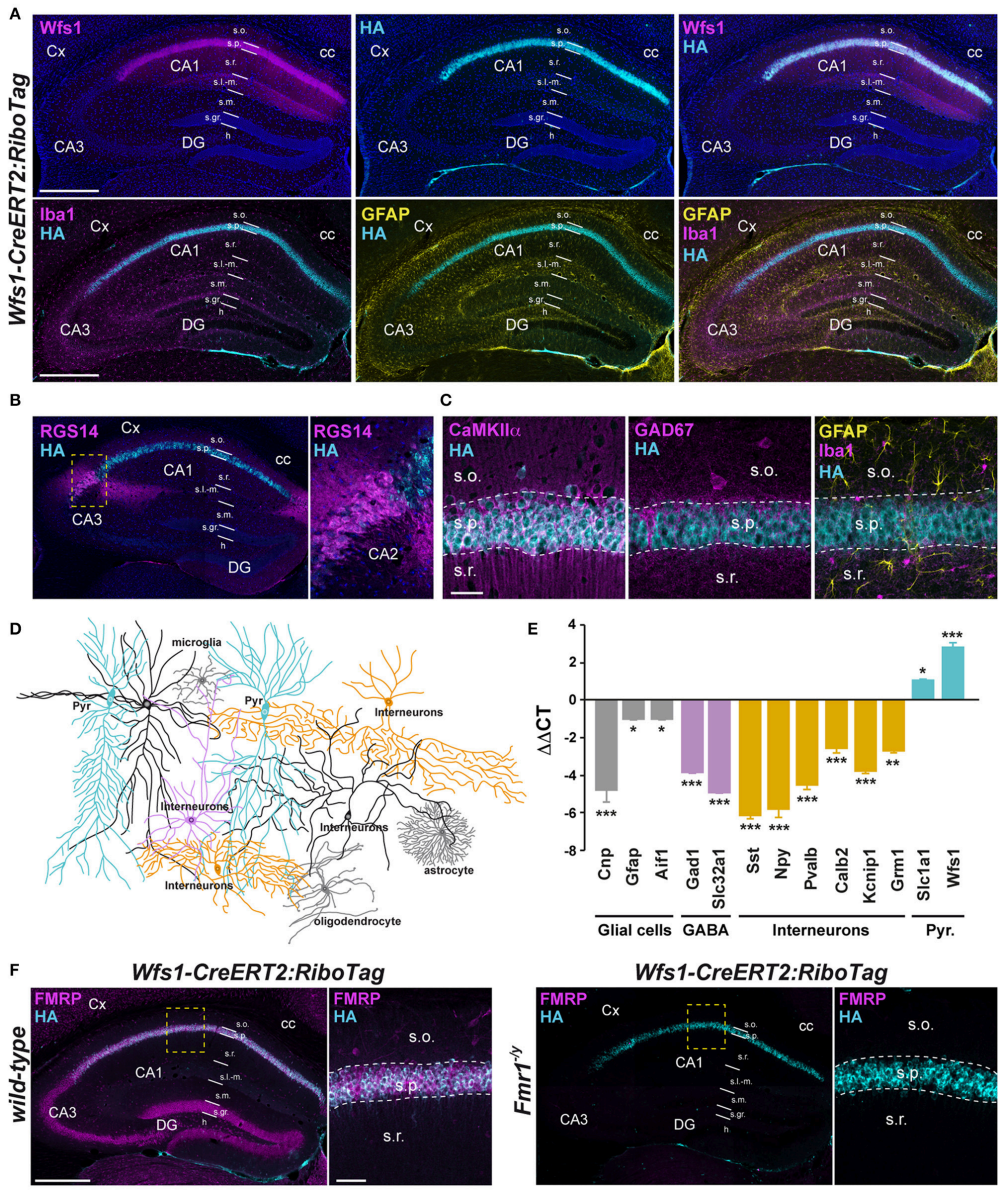
Characterization of *Wfs1-CreERT2:RiboTag:Fmr1*^−/*Y*^ mouse line. **(A)** Double immunofluorescence for Wfs1 (magenta) and HA (cyan) counterstained with DAPI (upper panels) and triple immunofluorescence for Iba1 (magenta), HA (cyan), and GFAP (yellow) (lower panels) in the dorsal hippocampus of *Wfs1-CreERT2:RiboTag* mice. Scale bars: 400 μm. **(B)** Double immunofluorescence for RGS14 (magenta) and HA (cyan). The lack of co-localization indicates that HA is preferentially expressed in CA1 but not CA2 pyramidal cells in *Wfs1-CreERT2:RiboTag* mice. **(C)** Triple immunofluorescence for HA (cyan), CaMKIIα (magenta), GAD67 (magenta), Iba1 (magenta), and GFAP (yellow) in the CA1 area of the dorsal hippocampus of *Wfs1-CreERT2:RiboTag* mice. Scale bars: 20 μm. For all immunofluorescence analyses, three slices per mouse were used (*n* = 3–4 mice/staining). **(D)** Drawing illustrating HA expression (cyan) among the distinct CA1 hippocampal cell types. **(E)** Validation by qRT-PCR (ΔΔCT) of the enrichment of pyramidal cell markers (*Slc1a1* and *Wfs1*; cyan bars) and de-enrichment of GABAergic (*Gad1, Slc32a1*; magenta bars, *Sst, Npy, Pvalb, Calb2, Kcnip1*, and *Grm1*; orange bars) and mossy cell markers (*Calb2*; orange bars) as well as glial cells markers (*Gfap, Cnp*, and *Aif1*; gray bars) after HA-immunoprecipitation from hippocampi of *Wfs1-CreERT2:RiboTag* mice. Data are expressed as the fold change comparing the pellet fraction vs. the input containing the mRNAs from all cellular types (*n* = 6 mice/genotype). **(F)** Double immunofluorescence for HA (cyan) and FMRP (magenta) in the dorsal hippocampus of wild-type (left panels) and *Fmr1*^−/*y*^ (right panels) *Wfs1-CreERT2:RiboTag* mice (*n* = 3 mice). Scale bars: 400 μm. High magnification images in CA1 subfield correspond to areas delineated by the yellow stippled squares. Scale bars: 50 μm. Cx, cortex; DG, dentate gyrus; cc, *corpus callosum*; s.o., *stratum oriens*; s.p., *stratum pyramidale*; s.r., *stratum radiatum*; s.l.-m. *stratum lacunosum-moleculare*; s.m, *stratum moleculare*; s.gr, *stratum granulosum*; h, *hilus*. ^*^p < 0.05; ^**^p < 0.01; ^***^p < 0.001.

To determine the identity of any differentially translated mRNAs linked to the loss of FMRP in CA1 pyramidal neurons, we generated an *Fmr1*^−/*y*^ mouse line allowing us to perform the RiboTag approach (Sanz et al., 2009). For this purpose, we first generated a double transgenic mouse line, by crossing the RiboTag mouse line (Sanz et al., 2009) with the tamoxifen-inducible *Wfs1-CreERT2* mouse line (Madisen et al., 2010) leading to the expression of the ribosomal protein Rpl22 tagged with the hemagglutinin (HA) epitope exclusively in Wolfram syndrome 1 (Wfs1)-expressing neurons (Luuk et al., 2008) (Figures 1, 2A). Double immunofluorescence analyses confirmed that HA expression was restricted to CA1 pyramidal cells (CaMKIIα) and absent from CA2 pyramidal cells (RGS14), GABAergic cells (GAD67), astrocytes (GFAP), and microglia (Iba1) (Figures 2A–C). As expected, expression of *Wfs1* and the glutamatergic marker *Slc1a1* was enriched after HA-immunoprecipitation on whole hippocampal lysates compared to the input fraction (which comprises all mRNAs from the initial homogenate) (Figures 2D,E). By contrast, gene expression markers for GABAergic cells (*Gad1, Slc32a1*), oligodendrocytes (*Cnp*), astrocytes (*Gfap*), microglia (*Aif1*), as well as interneuron-specific markers (*Sst, Npy, Pvalb, Calb2, Kcnip1, Grm1*) were all de-enriched (Figure 2E), validating the *Wfs1-CreERT2:RiboTag* mouse line to perform mRNA profiling in CA1 pyramidal cells. We therefore generated a triple transgenic mouse line (*Wfs1-CreERT2:RiboTag:Fmr1*^−/*y*^) (Supplemental Figure S1) allowing to assess the impact of the loss of FMRP (Figure 2F) on the translatome of CA1 pyramidal cells.

**Figure 3:**
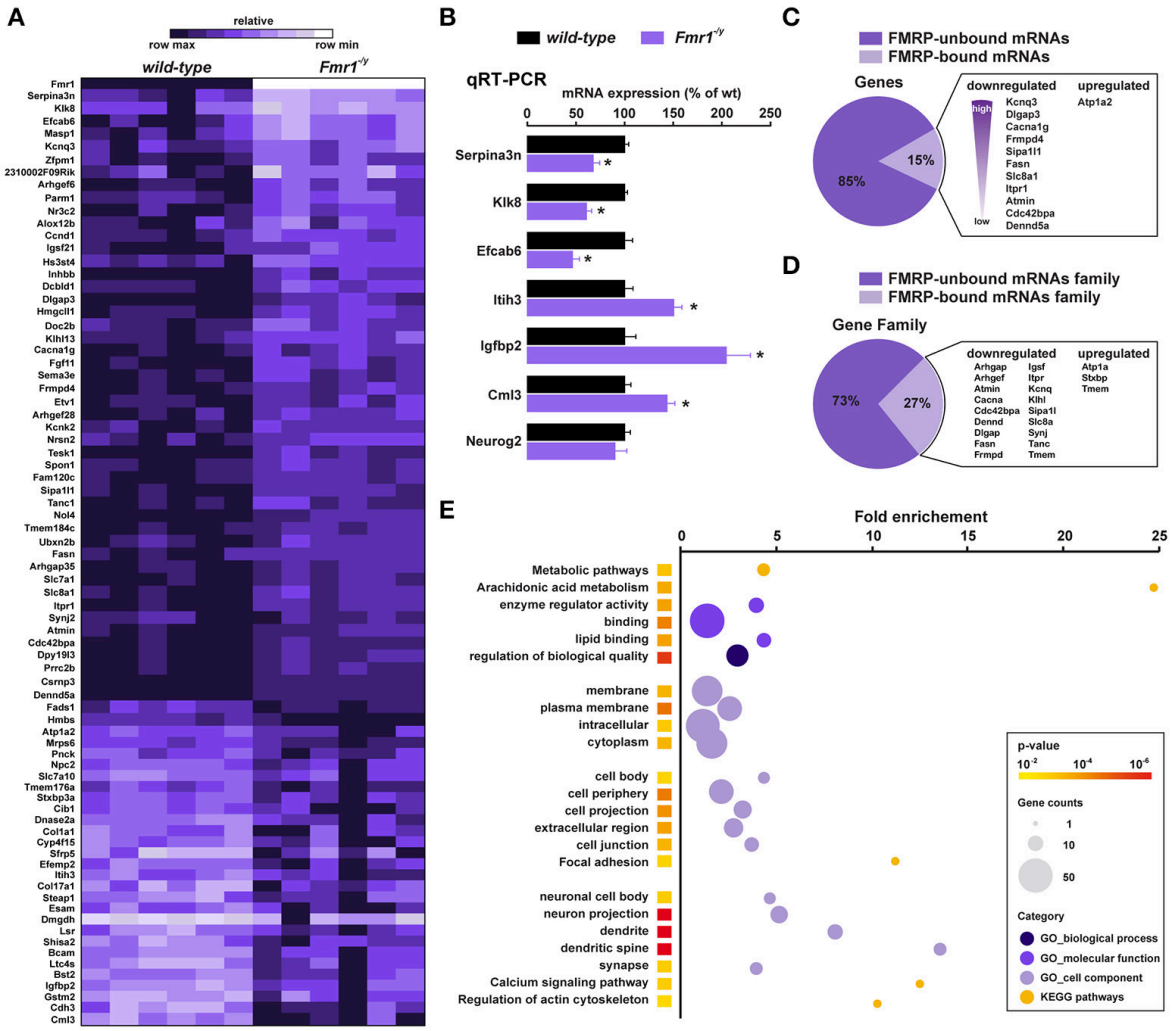
Translatome profile of CA1 pyramidal neurons in wild-type and *Fmr1*^−/*y*^ mice. **(A)** Heatmap of the 78 differentially regulated genes in CA1 pyramidal neurons between wild-type littermates and *Fmr1*^−/*y*^ mice. Scaled expression values are color-coded according to the legend (6 biological replicates/genotype, *n* = 4–6 mice/replicate). **(B)** Relative mRNA expression obtained by qRT-PCR analysis after HA immunoprecipitation of hippocampi from wild-type and *Fmr1*^−/*y*^ mice. The mRNA expression of each gene was normalized to the expression of the housekeeping gene β-actin. Results are represented as mean ± SEM (*n* = 6 mice/genotype). **(C,D)** Cross-analysis of our RNAseq data with FMRP-bound mRNAs **(C)** and mRNAs family **(D)** previously identified from mouse brain polyribosomes. **(E)** Gene Ontology (GO) analysis including Biological processes (darkened purple), Molecular function (purple) and Cellular component (lightened purple), and KEGG pathway (orange). The fold-enrichment is displayed for each GO term with Benjamini-Hochberg-corrected *p* values < 0.01. The size of the dots is proportional to the number of genes associated with a given GO term.

We next used high-throughput RNA sequencing (RNAseq) to perform genome-wide analyses of CA1 tagged ribosome-bound mRNAs in *Fmr1*^−/*y*^ mice and wild-type littermates (GEO; http://www-ncbi-nlm-nih-gov.insb.bib.cnrs.fr/geo/). Using a *P value* for a FDR < 0.05, our analysis identified 78 genes whose expression was different between the two genotypes. Among them, 49 genes were significantly downregulated and 29 upregulated (Figure 3A). Differentially expressed genes including downregulated (*Serpina3n, Klk8, Efcab6*), upregulated (*Itih3, Igfbp2, Cml3*) as well as equally expressed (*Neurog2*) genes were confirmed by qRT-PCR (Figure 3B), validating the reliability of gene quantification by the RNAseq approach. To gain insight into the nature of the differentially translated mRNAs in CA1 pyramidal neurons, we compared our data with the 842 FMRP-bound mRNAs previously identified from mouse brain polyribosomes (Darnell et al., 2011). Our cross-analysis revealed that 15% (12 out of 78) of these genes were FMRP-bound mRNAs. Given that 4.7% of the whole genome encodes for FMRP-binders (842 out of 17,823) this comparative analysis highlights a three-fold enrichment in FMRP-binders in our screen. Interestingly, among the 12 FMRP-bound mRNAs identified, 11 were downregulated (*Kcnq3, Dlgap3, Cacna1g, Frmpd4, Sipa1l1, Fasn, Slc8a1, Itpr1, Atmin, Cdc42bpa, Dennd5a*), while only *Atp1a2* was upregulated in *Fmr1*^−/*y*^ mice compared to wild-type littermates (Figure 3C). In addition, our study also revealed altered mRNAs translation of new sets of genes belonging to gene families known to be the target of FMRP (Figure 3D). Furthermore, 85% (66 out of 78) of the differentially translated mRNAs identified were FMRP-unbound mRNAs, suggesting that the loss of FMRP has a more widespread impact on mRNAs translation (Figure 3C). Together, these results reveal that FMRP controls the translation as well as the repression of a subset of mRNAs in CA1 pyramidal neurons.

To determine whether differentially translated mRNAs belong to specific physiological categories, we next performed Gene Ontology (GO) analysis, which included Biological Process, Cellular Component, and Molecular Function, as well as KEGG pathways analyses to map molecular interaction and reaction networks (Figure 2E). Notably, the most enriched GO categories comprised terms related to spine formation/maturation including synapses, dendritic spines, actin cytoskeleton, cell junctions and extracellular matrix (ECM) regulation (Figure 3E, Supplemental Figures S2–S6, Supplemental Table 3), suggesting a potential contribution in the aberrant spine maturation described in CA1 pyramidal neurons (Bilousova et al., 2009; He and Portera-Cailliau, 2013).

**Figure 4:**
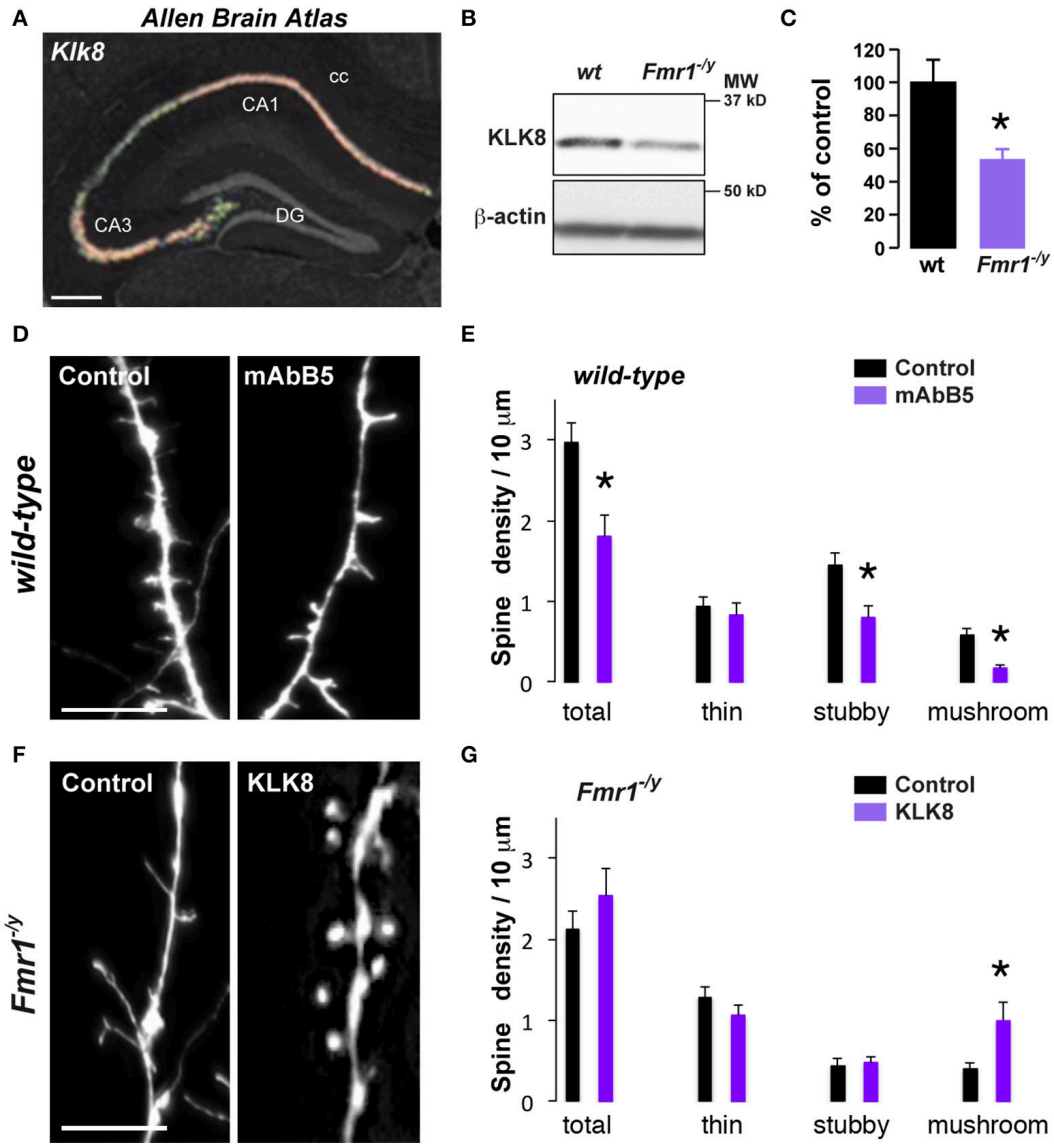
Decreased *Klk8* expression in *Fmr1*^−/*y*^ mice alters hippocampal dendritic spine maturation**. (A)** ISH coronal section from *Allen Brain Atlas* showing the enrichment of *Klk8* in the dorsal hippocampus. **(B)** WB of KLK8 in hippocampal homogenates of wild-type (wt) and *Fmr1*^−/*y*^ mice. **(C)** Quantification of KLK8 expression expressed as percentage of KLK8 expression in wild-type (wt) mice (*n* = 6 mice/genotype). **(D)** Red fluorescence from wt mice hippocampal primary culture (DIV14) incubated (mAbB5) or not (control, μg/35 mm dishes) with the activity-neutralizing anti-KLK8 antibody. Scale bar: 10 μm. **(E)** Quantification of spines density. **(F)** Red fluorescence from *Fmr1*^−/*y*^ mice hippocampal primary culture (DIV14) transfected with DsRed alone (control) or DsRed and KLK8-Venus (KLK8). Scale bar: 10 μm. **(G)** Quantification of spine density in *Fmr1*^−/*y*^ hippocampal neurons transfected or not with KLK8. Spine density **(E,G)** was quantified on three dendritic areas/neuron, 3–5 neurons/primary culture, and reproduced at least in 3 independent experiments per condition. DG, dentate gyrus; cc, *corpus callosum*. ^*^p < 0.05.

One key process in controlling structural synaptic plasticity is the ECM regulation. In particular, ECM proteases activity regulates cell adhesion at synapses and defects in proteases expression or function have been linked to FXS (Gkogkas et al., 2014; Sidhu et al., 2014). Interestingly, KLK8, a protease from the ECM displayed the most significant differential expression in our RNAseq analysis (just after *Fmr1*) together with *Serpina3n*, a serine-protease inhibitor regulating KLK8 activity, among other targets. Hence, to test for a causal link between mRNA dysregulation and impaired spine morphology in *Fmr1*^−/*y*^ mice, we selected the *Klk8* gene as candidate (Figure 3A). Its mRNA down-regulation in CA1 pyramidal cells from *Fmr1*^−/*y*^ mice was confirmed by qRT-PCR (Figure 3B). In wild-type mice, *Klk8* gene encodes for a trypsin-like serine protease exclusively expressed in limbic areas, with the highest expression in pyramidal neurons of the hippocampal CAl-CA3 subfields and to a lesser extent CA2 (Chen et al., 1995) (Figure 4A, http://mouse.brain-map.org). The analysis at the protein level by Western blot performed on hippocampal homogenates confirmed the decreased expression in *Fmr1*^−/*y*^ mice (Figures 4B,C). Upon neuronal activity, KLK8 triggers proteolytic cleavage of cell adhesion molecules (CAM), a mechanism contributing to dendritic spine maturation and stability (Matsumoto-Miyai et al., 2003). The blocking of KLK8 by a neutralizing antibody (mAbB5) resulted in a concomitant reduction in neuronal activity-induced L1-CAM processing and the amplitude of Schaffer collateral LTP (Komai et al., 2000; Matsumoto-Miyai et al., 2003). We therefore tested whether the density and the morphology of dendritic spines were affected by KLK8 activity. Notably, a significant reduction of the proportion of mature stubby- and mushroom-shaped spines was observed after application of the activity-neutralizing anti-KLK8 antibody (mAbB5) on primary culture of wild-type hippocampal neurons (Figures 4D,E). Because these structural changes are reminiscent of the immature protrusion phenotype observed in *Fmr1*^−/*y*^ mice (Figure 4F), we next assessed whether low levels of KLK8 might contribute to dendritic spine defects resulting from FMRP loss. Spine density and morphology were analyzed in FMRP-deficient neurons re-expressing KLK8 or not. As previously reported, hippocampal neurons cultured from *Fmr1*^−/*y*^ mice develop more immature spines (Figures 4F,G). In contrast, a significant increase in the number of mushroom-shaped spines was found in FMRP-deficient neurons re-expressing KLK8 (Figures 4F,G). Together these results demonstrate that KLK8 activity contributes to dendritic spine maturation and that aberrant synaptogenesis observed in *Fmr1*^−/*y*^ mice relies in part on altered levels of KLK8.

## Discussion

Enhanced global mRNA translation has been previously reported in the hippocampus of *Fmr1*^−/*y*^ mice (Qin et al., 2005; Dolen et al., 2007; Osterweil et al., 2010). Hence, numerous efforts have converged these last years in understanding which specific set of mistranslated mRNAs in absence of FMRP could cause neurological deficits including learning disabilities and cognitive impairment. A classical approach has consisted in the identification, by crosslinking immunoprecipitation, of FMRP interactions with rodent brain polyribosomal mRNAs, assuming that FMRP binders could play important functions in synaptic plasticity. Although studies efficiently used this strategy to identify gene candidates relevant to synaptic functions (Darnell et al., 2005, 2011; Tabet et al., 2016), this method was restricted to FMRP targets, ignoring the functional importance of mRNAs that do not bind FMRP. By opposition and without assumption, in the present work we identified genes whose expression was modified in the *Fmr1*^−/*y*^ adult mice compared to control mice, as a snapshot integrating all dysregulated mRNAs. Interestingly, our study shows that the loss of FMRP has a widespread impact on mRNAs translation since both FMRP-bound and unbound mRNAs are differentially translated. These expression changes are restricted to a subset of 78 genes. Noteworthy, both up- and down-regulation of genes are important to fully comprehend the ethiology of this neurodevelopmental disorder.

Our study provides evidence about the molecular consequences associated with FMRP loss. We show that 15% of the mRNAs differentially regulated in *Fmr1*^−/*y*^ compared to control mice are FMRP-binders. Hence, the proportion of transcripts that bind FMRP in this list of genes differentially regulated is three-fold enriched compared to their proportion in the whole genome, comforting the function of FMRP as direct regulator of mRNA translation. In absence of FMRP, all but one of the identified FMRP-bound mRNAs were down-regulated. This result is consistent with previous data suggesting that FMRP would inhibit translation by stalling ribosomal translocation on its mRNA interactors, increasing their accumulation (although not translated) at synapses (Darnell et al., 2011). Furthermore, a bi-functional role of FMRP has been recently proposed to suppress or de-repress its target transcripts through the miRNA pathway by modulating RNA secondary structure (Kenny and Ceman, 2016). In our study, the majority of FMRP-binders were not found to be differentially regulated in CA1 pyramidal cells from adult mice hippocampi, suggesting a possible dysregulated expression during neurodevelopment. Finally, our data also highlights the preponderance (85%) of non-FMRP binders among the genes differentially regulated between adult *Fmr1*^−/*y*^ and control mice. The subset of altered translated mRNAs found herein, indirectly linked to the absence of FMRP and consecutive to neurodevelopmental adaptations, expands the list of pertinent gene candidates that could counteract neuronal plasticity defects, learning disabilities and cognitive impairments of FXS.

Analyzing gene functions, we further demonstrated that those differentially translated mRNAs belong to specific physiological categories, including synapses, dendritic spines, calcium signaling, actin cytoskeleton, cell junctions, and extracellular matrix regulation, together supporting connectivity-related processes. Calcium signaling is critical for induction of excitatory synaptic plasticity. FXS models display both calcium transient and calcium store release defects (Tessier and Broadie, 2011). This unreliable calcium signaling causes plasticity alterations (Meredith et al., 2007). Here, we identified genes related to calcium signaling that might be involved in plasticity-related disorders. Thus, we found that *Itpr1* (IP3 receptor 1), which controls calcium release from intracellular stores and is an autism spectrum disorder-associated gene (Xu et al., 2012), is dysregulated in CA1 pyramidal neurons of *Fmr1*^−/*y*^ mice. Our RNAseq also revealed the *Slc8a1* and *Cacna1g* genes, which code for a sodium/calcium exchanger and a voltage-dependent T-type calcium channel subunit respectively, and are both found to be mis-expressed in human neural progenitor cells from FXS patients (McMillan et al., 2012). Dysregulation of these three genes might thus participate to aberrant functional plasticity phenotypes. Alteration of calcium signaling dynamics in FXS models also suggests that activity-driven calcium signaling would couple structural and functional developmental changes in the FXS disease state (Doll and Broadie, 2014). Consistent with a role on spine morphology, we also identified genes involved in regulation of actin cytoskeleton such as *Arhgef6* (Rac/Cdc42 guanine nucleotide exchange factor 6), *Fgf11* (fibroblast growth factor 11), and *Grlf1* (glucocorticoid receptor DNA binding factor 1) which could be responsible for elevated density of elongated spines in FXS patients (Irwin et al., 2001).

Dendritic spine defects resulting from FMRP loss have also been well-documented in mice (Comery et al., 1997; Portera Cailliau and Yuste, 2001). Our study shows a prevalence of immature spines in cultured hippocampal neurons from *Fmr1*^−/*y*^ mice compared to control mice, in agreement with previous data suggesting that the most consistent trouble with spines in FXS would be a deficiency in activity-dependent spine plasticity and maturation (He and Portera-Cailliau, 2013). Among structural plasticity-related functions, genes expressed at the ECM, linking pre- and postsynaptic elements, are of major interest. In particular, neural ECM proteases control learning and synaptic plasticity (Tsilibary et al., 2014). We found that in absence of FMRP, *Klk8*, a gene coding for an ECM serine protease highly expressed in CA1 pyramidal cells of the hippocampus (Chen et al., 1995), displayed one of the most significant changes in gene expression. Importantly, we show that reduced levels of KLK8 in CA1 pyramidal neurons contribute to the abnormal spine morphology observed in *Fmr1*^−/*y*^ mice by preventing the maturation of mushroom-shaped spines, further supporting the role of proteins involved in ECM regulation, such as the metalloproteinase 9 in the dendritic spine abnormalities associated with FMRP loss (Janusz et al., 2013; Gkogkas et al., 2014; Sidhu et al., 2014). In addition to its role in structural plasticity, KLK8 has also been reported to play a role in synaptic plasticity in the CA1 subfield as well as in some hippocampus-dependent learning (Tamura et al., 2006; Ishikawa et al., 2011). The loss of KLK8 has also been shown to predispose to seizures (Davies et al., 2001). Future studies will determine whether altered KLK8 expression also participates in the impaired synaptic plasticity, cognitive deficits and epileptiform discharges observed in *Fmr1*^−/*y*^ mice.

By using a cell-type specific RNAseq approach, our study shows that not only are both FMRP-bound and unbound mRNAs differentially translated, but also that this altered translation is bidirectional and restricted to a subset of genes. These findings are in agreement with a recent study describing a cell-type-specific translation profiling in juvenile *Fmr1*^−/*y*^ mice, using a BAC-TRAP strategy where the cholecystokinin (Cck) promoter drives EGFP-L10a expression (Thomson et al., 2017). Interestingly, the translatome profile identified by Thomson et al. strongly differs from ours. Several experimental conditions could explain this apparent discrepancy: the age of mice (25–32 days vs. 2–6 months), their genetic background (hybrid FVB × C57BL/6J vs. C57BL/6J mice), the promoter-driven expression of the tagged ribosomal subunit (Cck vs. Wfs1), the sequencing depth (50 or 75 bp, paired end vs. 50 bp single read) as well as the default false discovery rate (FDR) chosen for gene analyses (DESeq2 at the FDR of 0.1 vs. 0.05). Moreover, since heterogeneous ribosomes preferentially translate distinct subpools of mRNAs (Shi et al., 2017), the nature of the tagged ribosomal subunit (Rpl10a vs. Rpl22) has also to be carefully taken into consideration while performing translation profiling.

To conclude, our study identified gene candidates involved in neuronal connectivity-related functions, which should together support hippocampus-dependent processes. These genes could be new therapeutic targets to rescue physiological neuronal plasticity in FXS.

## Methods

### Animals

Adult (2–6 month) C57BL/6J mice were used in this study. The mouse breeding strategy is described in Figure S1. Mice were treated for 3 days with tamoxifen (100 mg/kg, i.p., Sigma), dissolved in sunflower oil/ethanol (10:1) to a final concentration of 10 mg/ml, and used 1 week later for immunostaining or immunoprecipitation studies. Animals were housed under standardized conditions with a 12 h light/dark cycle, stable temperature (22 ± 2°C), controlled humidity (55 ± 10%), and food and water *ad libitum*. All experiments were in accordance with the guidelines of the French Agriculture and Forestry Ministry for handling animals (D34-172-13).

### Immunofluorescence

Tissue preparation and immunofluorescence were performed as described (Biever et al., 2015). Briefly, free-floating sections were rinsed in Tris-buffered saline (TBS; 0.25 M Tris and 0.5 M NaCl, pH 7.5), incubated 15 min in 0.2% Triton X-100 in TBS, and blocked for 1 h in 3% bovine serum albumin (BSA) in TBS. Slices were then incubated in 0.15% Triton X-100 and 1% BSA in TBS overnight at 4°C with the primary antibodies listed in Supplemental Table S1. The following day, slices were rinsed three times in TBS and incubated 45 min with goat Cy3-coupled anti-rabbit or anti-chicken (1:500; Jackson ImmunoResearch Laboratories), goat Cy5-coupled anti-rabbit (1:500; Jackson ImmunoResearch Laboratories), and goat Alexa Fluor 488-coupled anti-mouse (1:500; Invitrogen) secondary antibodies. Sections were rinsed twice in TBS and twice in 0.25 M Tris-buffer before mounting in 1,4-diazabicyclo-[2.2.2]-octane (DABCO, Sigma-Aldrich). Three slices per mouse were used in all immunofluorescence analyses (*n* = 3–4 mice/staining).

Confocal microscopy and image analysis were carried out at the Montpellier RIO Imaging Facility. Images covering the entire dorsal hippocampus were single confocal sections acquired using sequential laser scanning confocal microscopy (LSM780; Zeiss). Photomicrographs were obtained with the following bandpass and long-pass filter setting: Alexa Fluor 488/Cy2 (bandpass filter: 505–530), Cy3 (bandpass filter: 560–615), and Cy5 (long-pass filter 650). All parameters were held constant for all sections from the same experiment.

### Polyribosome immunoprecipitation

Tagged ribosome immunoprecipitation was performed as described previously (Puighermanal et al., 2017) in the whole hippocampus of *Wfs1-CreERT2:RiboTag:Frm1*^−/*y*^ and wild-type mice. Total RNA was extracted from ribosome-mRNA complexes using RNeasy Microkit (Qiagen) followed by in-column DNAse treatment to remove genomic DNA contamination. Quality and quantity of RNA samples were both assessed using Agilent Bioanalyzer 2100 (Agilent Technologies). Six biological replicates, each one composed of a pool of 4–6 mice, were used for RNAseq analysis.

### cDNA synthesis and quantitative real-time PCR

After isolation of tagged ribosome-bound mRNAs, synthesis of cDNA and qRT-PCR were performed as previously described (Puighermanal et al., 2017) (*n* = 6 mice/genotype). The ΔΔCT (Δct1-Δct2) method was applied to quantify mRNA changes using β-actin or Hprt2 as housekeeping genes. In Figure 2, the immunoprecipitated RNA samples (pellet) were compared to the input samples, whereas in Figure 3 the immunoprecipitated RNA samples (pellet) from wild-type and Fmr1^−/y^ were compared. Primer sequences are indicated in Supplemental Table S2.

### Stranded mRNA library preparation and sequencing

Six biological replicates, each one composed of a pool of 4–6 mice, were analyzed by RNA Sequencing (RNAseq). RNAseq libraries were constructed with the Truseq stranded mRNA sample preparation (Low throughput protocol) kit from Illumina. One microgram of total RNA was used for the construction of the libraries. The first step in the workflow involves purifying the poly-A containing mRNA molecules using poly-T oligo attached magnetic beads. Following purification, the mRNA is fragmented into small pieces using divalent cations under elevated temperature. The cleaved RNA fragments are copied into first strand cDNA using SuperScript II reverse transcriptase, Actinomycin D and random hexamer primers. The Second strand cDNA was synthesized by replacing dTTP with dUTP. These cDNA fragments then have the addition of a single “A” base and subsequent ligation of the adapter. The products are then purified and enriched with 15 cycles of PCR. The final cDNA libraries were validated with a Fragment Analyzer (Advanced Analytical, Ankeny, IA) and quantified with a KAPA qPCR kit (Kapa Biosystems, Wilmington, MA). For each sequencing lane of a flowcell V4, four libraries were pooled in equal proportions, denatured with NaOH and diluted to 8 pM before clustering. Cluster formation, primer hybridization and single end-read 50 cycles sequencing were performed on cBot and HiSeq2500 (Illumina, San Diego, CA), respectively.

### Bioinformatic RNAseq analysis

Image analyses and base calling were performed using the Illumina HiSeq Control Software and Real-Time Analysis component. Demultiplexing was performed using Illumina's conversion software (bcl2fastq 2.17). The quality of the raw data was assessed using FastQC (v0.11.5) from the Babraham Institute and the Illumina software SAV (Sequencing Analysis Viewer). A splice junction mapper, TopHat 2.1.1 (Kim et al., 2013) (using Bowtie 2.2.8; Langmead and Salzberg, 2012), was used to align the RNAseq reads to the mouse genome (UCSC mm10) with a set of gene model annotations (genes.gtf downloaded from UCSC on May 23 2014). Final read alignments having more than three mismatches were discarded. Samtools (1.2) was used to sort the alignment files. Then, the counting was performed with HTSeq count 0.6.1p1 (union mode) (Anders et al., 2015). The data is from a strand-specific assay, the read has to be mapped to the opposite strand of the gene. Before statistical analysis, genes with <15 reads (cumulating all the analyzed samples) were filtered and thus removed. Differentially expressed genes were identified using the Bioconductor (Gentleman et al., 2004) package DESeq2 1.6.3 (Love et al., 2014). Data were normalized using the DESeq2 normalization method. Genes with adjusted *p* < 5% (according to the FDR method from Benjamini-Hochberg) were declared differentially expressed. To perform the functional analysis of the resulting list of genes with the Gene Ontology (GO) annotations, the topGO (Alexa et al., 2006) package from Bioconductor was used. Overrepresented GO terms were identified using Fisher's exact test with the weight method that is implemented in the topGO package. As confidence threshold we used a *P*-value of 1%. To perform this analysis the differentially expressed genes were compared with those of all known genes present in the annotation. The GO categories were found in the Org.Mm.eg.db package based on the gene reporter EntrezGeneID. The RNAseq data from this study have been submitted to the NCBI Gene Expression Omnibus GEO; http://www-ncbi-nlm-nih-gov.insb.bib.cnrs.fr/geo/ under accession number GSE94559.

### Hippocampal primary cell culture, plasmids, and transfection

pcDNA3.1-mKLK8-Venus plasmid was obtained by PCR amplification of the mouse Klk8 ORF from pCMV3-mKLK8-OFP Spark (Sino Biologicals MG50967-ACR) subcloned into pcDNA3.1 using HindIII/AgeI restriction sites. Dissociated-cell cultures of hippocampal neurons were prepared from E17.5 embryonic mice. Neurons were seeded on poly-ornithine coated coverslips and grown in Neurobasal medium supplemented with 2% B27 supplement, 10% fetal bovine serum, 0.5 mM glutamine, 0.25 mM glutamic acid and antibiotics (100 U/ml penicillin and 100 mg/ml streptomycin). After 3 days in culture, the medium was supplemented with 5 μM cytosine b -D-arabinofuranoside hydrochloride (Sigma) for 12 h. Half of the medium was then replaced with Neurobasal medium supplemented with 2% B27, 0.5 mM Glutamax and antibiotics. Wild-type hippocampal neurons were transfected with pDsRED-N1 (Clontech Laboratories, PT3725-5, 0.2 μg/12 mm dish) plasmid at 12 Days *In Vitro* (DIV12) using Lipofectamine 2000 (Invitrogen) according to the manufacturer's protocol. Neurons were treated at DIV14 for 1 h with the activity-neutralizing anti-KLK8 antibody (mAbB5 2.5 μg/ml; Medical and Biological Laboratories, Japan) or with PBS. They were then fixed in 4% paraformaldehyde and 4% sucrose in PBS for 20 min and washed three times with PBS. This specific inhibitor of KLK8 protease activity was previously characterized (Momota et al., 1998) and used for *in vitro* electrophysiological experiments (Matsumoto-Miyai et al., 2003). *Fmr1*^−/*y*^ mice hippocampal neurons were transfected at DIV12 with pDsRED-N1 and pcDNA3.1-Venus (control) or pDsRED-N1 and pcDNA3.1-mKLK8-Venus (0.2 μg of each plasmid/12 mm dish) and fixed at DIV14.

### Western blot analysis

Hippocampal tissue samples were homogenized in a lysis buffer containing 20 mM Tris pH 6.8, 137 mM NaCl, 2 mM EDTA, 1% Triton X-100, 10% Glycerol, 0.5 mM DTT, and protease inhibitors. Protein concentrations were determined by BCA protein assay (Sigma). Equal amounts of lysates were separated on a 12% SDS polyacrylamide gel (50 μg of total protein per lane) and transferred onto nitrocellulose membranes (GE Healthcare) at 40 V overnight at 4°C. After incubation for 1 h in blocking buffer (PBS, 0.1% Tween 20, and 5% dried non-fat milk), membranes were incubated for 2 h at room temperature with the primary antibodies listed in Supplemental Table S1. Secondary HRP-conjugated antibodies (Jackson ImmunoResearch Laboratories) were used at a 1:4,000 dilution and visualized by enhanced chemiluminescence detection (PerkinElmer). The immunoblot signals were acquired on a ChemiDoc Touch Imaging System (Bio-Rad). For quantification of changes in protein expression levels, band intensities were measured with ImageJ software. The optical density values of KLK8 were normalized to the detection of β-actin values in the same sample.

### Spine quantification

Images were acquired with an AxioimageZ1-apotome microscope (Zeiss filterset43: λ ex 545/25–λ em 605/70) and analyzed using Fiji software. According to previous publications (Peters and Kaiserman-Abramof, 1969) (and see Hering and Sheng, 2001 for review) dendritic spines have been classified by shape as thin, stubby, and mushroom-shaped. Spines were defined as dendritic protrusions smaller than 5 μm in length and subdivided in three standard morphological classes (thin spines: length ≥ 1 μm and head diameter < 0.4 μm; stubby: length < 1 μm; mushrooms: length ≥ 1 μm, and head diameter > 0.4 μm).

### Statistical analyses

GraphPad Prism v6.0 software was used for statistical analyses. Data are shown as the means ± SEM. We used Student's *t* test for normally distributed parameters and non-parametric Mann-Whitney test for small samples, where ^*^p < 0.05; ^**^p < 0.01 and ^***^p < 0.001.

## Author contributions

LC conceived and supervised the breeding strategy, designed, performed and analyzed all *in vitro* experiments. NB and EP performed biochemical and histological experiments. JV performed qRT-PCR. SR and DS performed the RNAseq and bioinformatic analyses. EV, JP, and EP conceived, designed, led the project and wrote the manuscript.

### Conflict of interest statement

The authors declare that the research was conducted in the absence of any commercial or financial relationships that could be construed as a potential conflict of interest.
