# Supplementary material for: Cell Type-Specific mRNA Dysregulation in Hippocampal CA1 Pyramidal Neurons of the Fragile X Syndrome Mouse Model
Source: Front Mol Neurosci. 2017 Oct 20;10:340. doi: 10.3389/fnmol.2017.00340 (PMC5655025; doi:10.3389/fnmol.2017.00340)
Supplement: Supplementary file 1 [file DataSheet1.PDF]

Supplemental materials

Cell type-specific mRNA dysregulation in hippocampal CA1 pyramidal neurons of the Fragile X syndrome mouse model.

Laura Ceolin[1], Nathalie Bouquier[1], Jihane Vitre-Boubaker[1], Stéphanie Rialle[2], Dany Severac[2], Emmanuel Valjent[1], Julie Perroy[1]*, Emma Puighermanal[1]*

Supplemental Figures

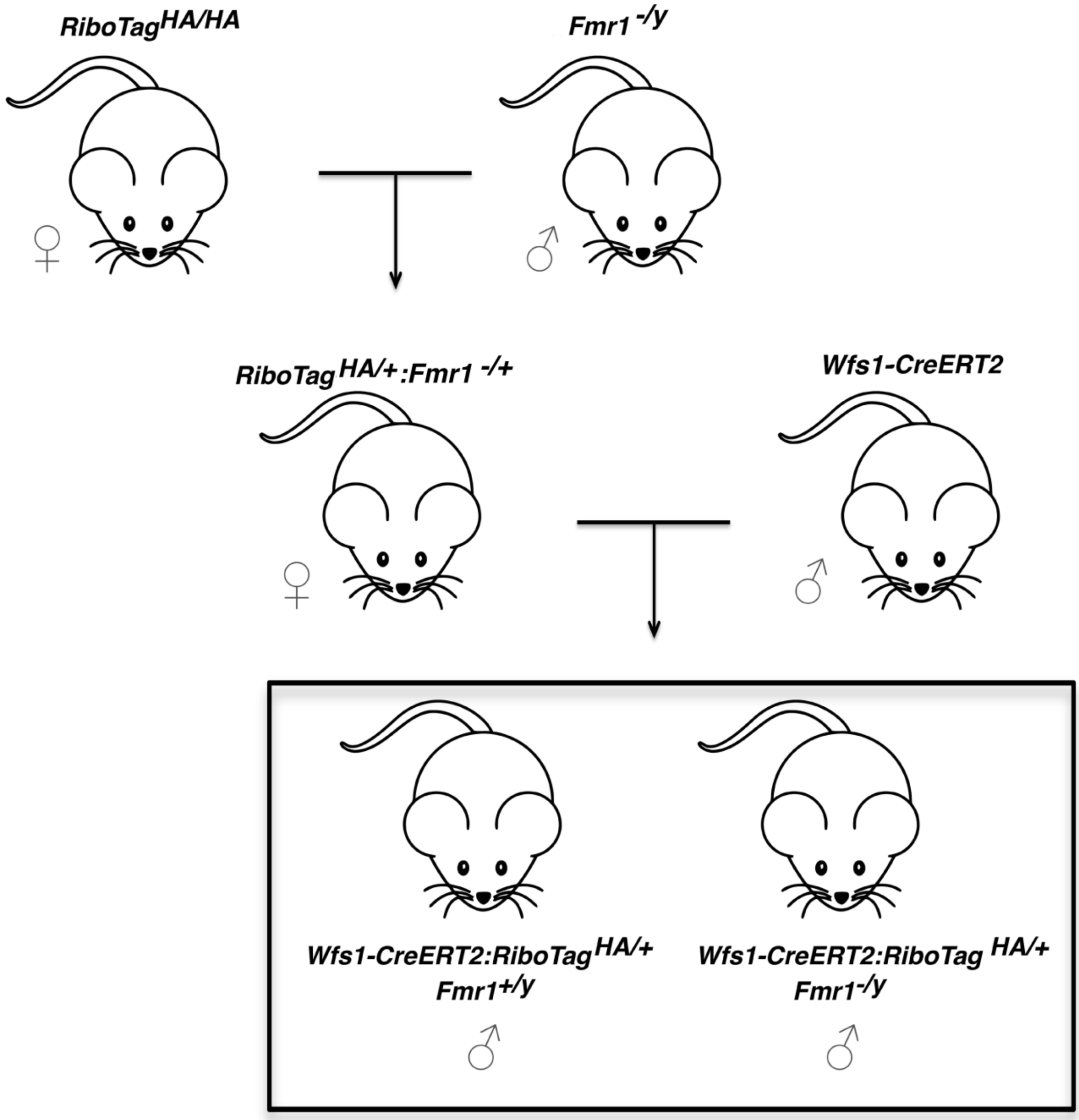

Figure S1. Scheme of breeding strategy.

RiboTag mice (homozygous *Rpl22*$^{HA/HA}$, strain B6N.129-Rpl22tm1.1Psam/J, The Jackson Laboratory) [1] were crossed to the *Fmr1*$^{-/y}$ males (Gift from Pr. Rob Willemsen [2]). Offspring females from RiboTag-Fmr1$^{-/+}$ were bred with *Wfs1-Tg3-CreERT2* BAC transgenic Cre mouse line (strain B6;C3-Tg[Wfs1-cre/ERT2]3Aibs/J, The Jackson Laboratory [3]) to produce the *Wfs1-CreERT2:RiboTag-Fmr1*$^{+/y}$ and *Wfs1-CreERT2:RiboTag-Fmr1*$^{-/y}$ males used in this study. Cre recombinase-expressing mouse results in deletion of the wild-type exon 4 in the Wfs cell population and replacement with the Rpl22HA exon 4. Both males and females Wfs1-CreERT2:Ribotag mice aged between 2-6 months were used for the

characterization of the mouse line. Only males aged between 2-6 months were used for RNAseq.

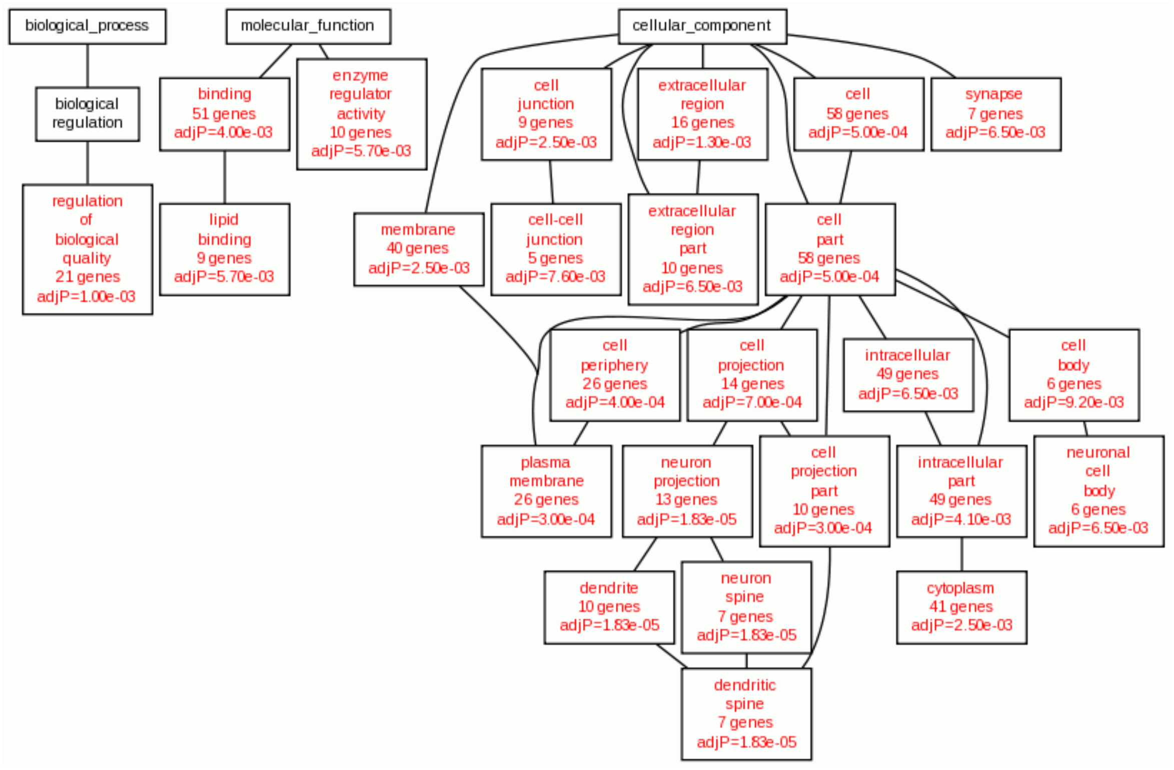

Supplemental Figure S2. Raw and exhaustive data from WEBGESTALT (http://bioinfo.vanderbilt.edu/webgestalt/) of GO terms enriched in the set of genes differentially regulated between wild type and *Fmr1*$^{-/y}$ mice.

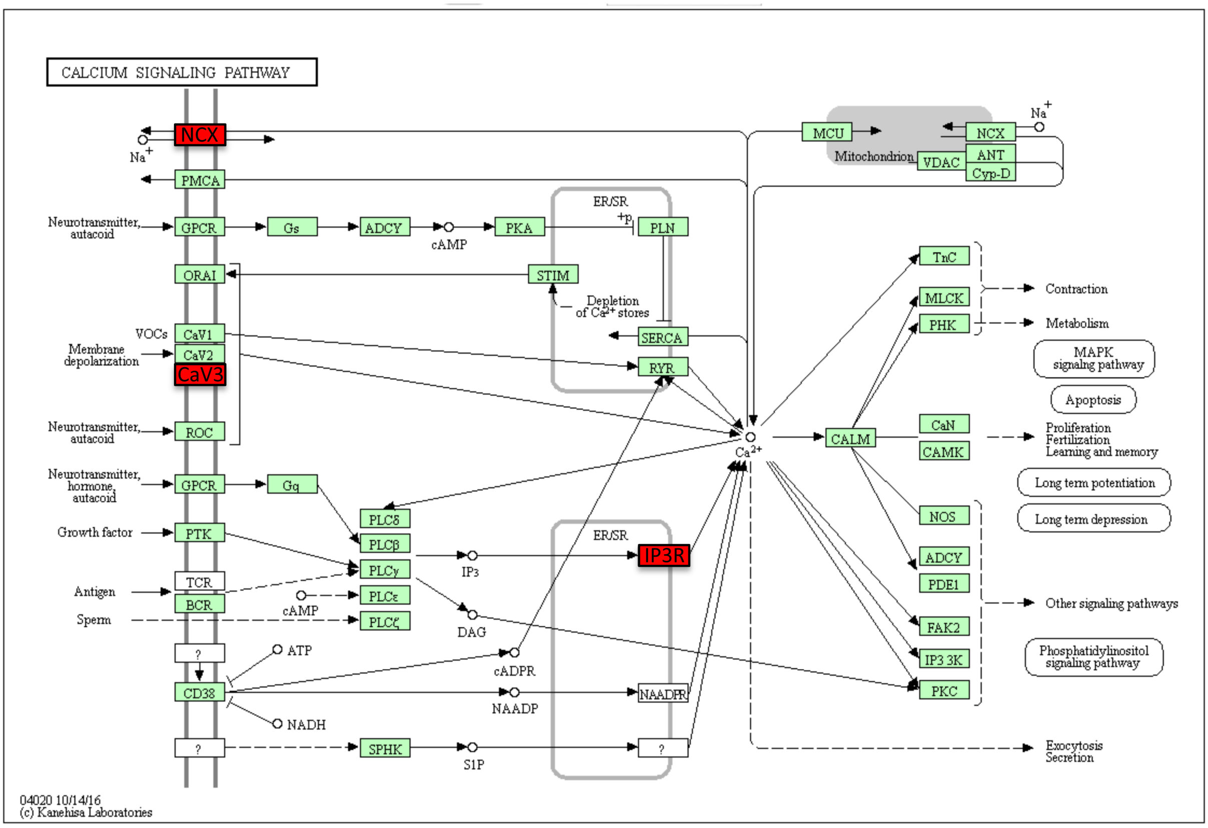

Supplemental Figure S3. Neuronal-plasticity related KEGG pathways enriched in the set of genes differentially regulated between wild type and *Fmr1*$^{-/y}$ mice. Referenced KEGG pathways were implemented to draw the involvement of these genes (red box) in specific molecular interaction and reaction networks. Calcium signaling pathways ; http://www.genome.jp/kegg-bin/show_pathway?mmu04020.

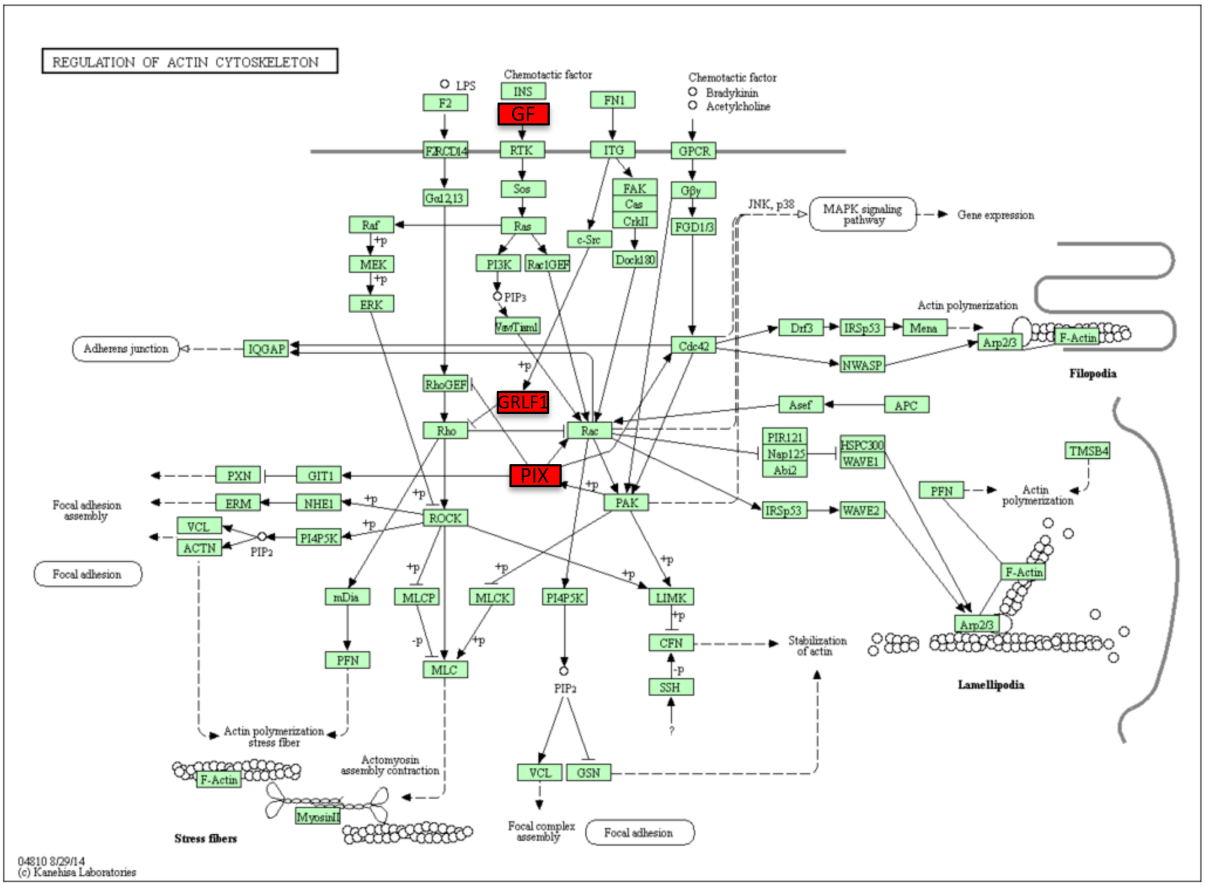

Supplemental Figure S4. Neuronal-plasticity related KEGG pathways enriched in the set of genes differentially regulated between wild type and *Fmr1*$^{-/y}$ mice. Referenced KEGG pathways were implemented to draw the involvement of these genes (red box) in specific molecular interaction and reaction networks. Regulation of actin cytoskeleton ; http://www.genome.jp/kegg-bin/show_pathway?mmu04810

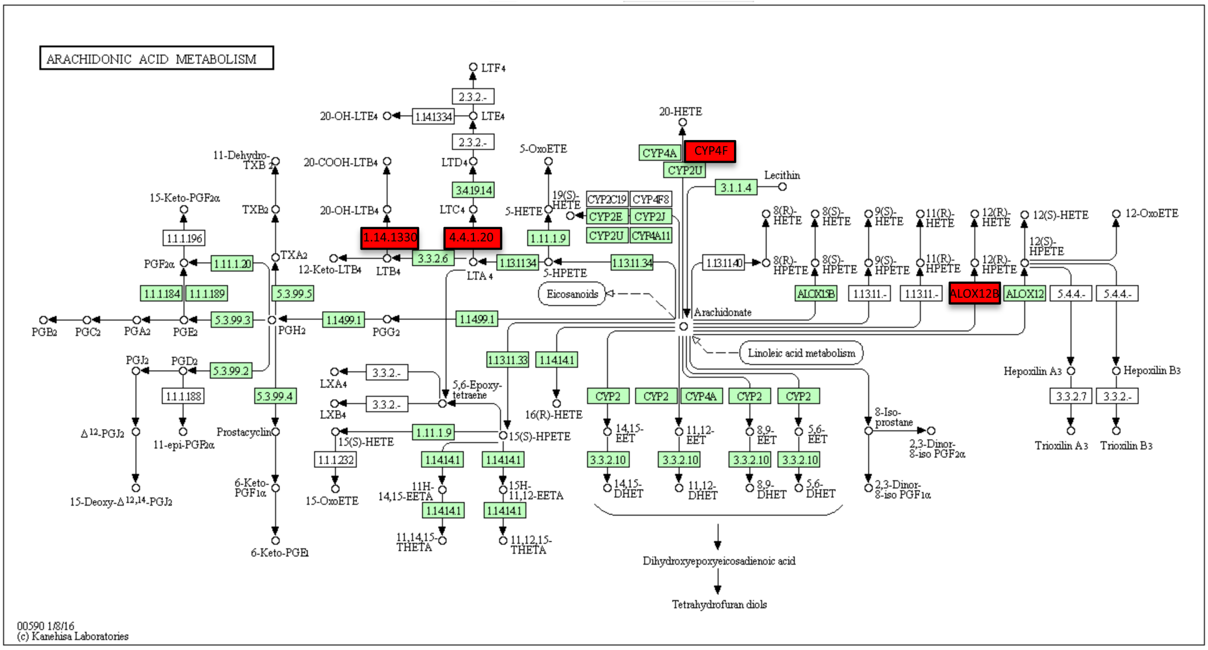

Supplemental Figure S5. Neuronal-plasticity related KEGG pathways enriched in the set of genes differentially regulated between wild type and *Fmr1*$^{-/y}$ mice. Referenced KEGG pathways were implemented to draw the involvement of these genes (red box) in specific molecular interaction and reaction networks. Arachidonic Acid metabolism ; http://www.genome.jp/kegg-bin/show_pathway?mmu00590

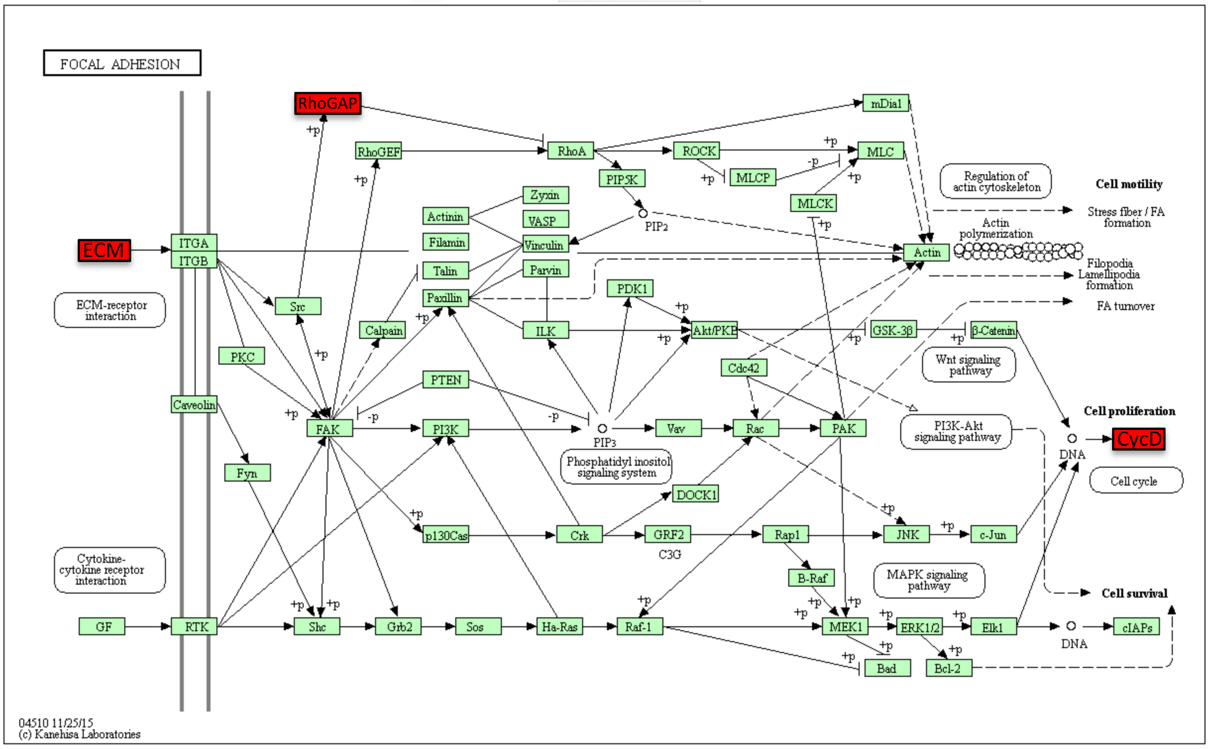

Supplemental Figure S6. Neuronal-plasticity related KEGG pathways enriched in the set of genes differentially regulated between wild type and *Fmr1*$^{-/y}$ mice. Referenced KEGG pathways were implemented to draw the involvement of these genes (red box) in specific molecular interaction and reaction networks. Focal adhesion ; http://www.genome.jp/kegg-bin/show_pathway?mmu04510.

Supplemental Tables

Supplemental Table S1: List of Primary Antibodies

Antigen	Species	Dilution	Supplier/Catalog no./References
Wfs1	Rabbit	1:500 (IF)	[4]
HA	Mouse	1:1000 (IF)	Covance (#MMS-101R)
HA	Rabbit	1:1000 (IF)	Rockland (600-401-384)
c-Myc	Mouse	1:180 (IP)	ThermoFisher (MA1-980)
Iba1	Rabbit	1:500 (IF)	Wako (019-19741)
GFAP	Chicken	1:1000 (IF)	Abcam (ab4674)
GAD67	Mouse	1:1000 (IF)	Millipore (mab5406)
CaMKIIα	Mouse	1:1000 (IF)	Millipore (05-532)
FMRP	Rabbit	1: 500 (IF)	Millipore (ab60-46)
RGS14	Mouse	1:1000 (IF)	NeuroMab (75-170)
KLK8	Rabbit	1: 500 (WB)	Santa Cruz (sc-292341)
β-actin	Mouse	1: 5000 (WB)	Sigma (A5441)

Supplemental Table S2: Sequences of PCR primers

Genes	PCR primers	
	Forward	*Reverse*
Cnp	GCTGCACTGTACAACCAAATTCTG	ACCTCCTGCTGGGCGTATT
Gfap	AGCGAGCGTGCAGAGATGA	AGGAAGCGGACCTTCTCGAT
Aif1	CCCCCAGCCAAGAAAGCTAT	GCCCCACCGTGTGACATC
Gad1	TTGTGCTTTGCTGTGTTTTAGAGA	CCCCCTGCCCAAAGATAGAC
Slc32a1	TCACGACAAACCCAAGATCAC	GTCTTCGTTCTCCTCGTACAG
Sst	CTGTCCTGCCGTCTCCAGTG	CTCTGTCTGGTTGGGCTCGG
Npy	GCTCTGCGACACTACATCAA	GGCGTTTTCTGTGCTTTCCT
Pvalb	TCGATGACAGACGTGCTCAG	CTTCACCTCATCCGGGTTCT
Calb2	TGAGAATGAACTGGACGCCCTC	GTAGAGCTTCCCTGCCTCGG
Kcnip1	GCGGTGTGGTCAATGAAGAA	GTCGAAGGCATTGAAGAGGT
Grm1	TTCAAGACCCGCAACGTGCC	CAGACTTGCCGTTAGAATTGG
Slc1a1	AAAGATAGCAGGAAGGTAACCGAAT	CGGTCAGTCGGTAGCTTTCAG
Wfs1	TGCTCACCTGTAGATCGCAGAT	CTTATGACACTCGGCATTCAAAGT
Fmr1	GTCAGGAGTTGTGAGGGTGA	GGTCCAACCCTTGAATTATTGGA
Serpina3n	GGATGTCCTTTCAAAGCTGGGCAT	GGGACAAATTTGACTCCAGTGGCAG
Klk8	TTCCCAGAACAAGTGTGAGAGAGC	GTAGACTCCAGGTTTCTCGGGTTTC
Efcab6	GCGTTCCTTCAAGACCTACGACAAG	GAAGGCACGCAGGAAGTCATTGTAG
Itih3	GCTCTGACCCTACAAAGCCAGATG	CAAACCAGCAGATGACCTTGGTG
Igfbp2	TCCCGAACACCAGCAGAAAT	AATACATGTGTCAAAACTGGGAACTC
Cml3	GATTGAGGCCTGTGTTCCTGAAAGC	GAGTCAAGTAGATGGGCTGTGGAGA
Neurog2	ATGCACAACCTAAACGCCGC	CGGTGAGCGCCCAGATGTAA

Supplemental Table S3:

List of genes by function. The results for each enriched GO category and KEGG pathways are listed in this table. For each category, the first (bold) row lists its sub-root, category name, and corresponding GO / KEGG ID. The second row lists number of reference genes in the category (C), number of genes in the gene set and also in the category (O), expected number in the category (E), Ratio of enrichment (R), p value from hypergeometric test (rawP), and p value adjusted by the multiple test adjustment (adjP). Finally, genes in the category are listed. For each gene, the table lists the Gene symbol and description.

**cellular comp**	**DENDRITIC SPINE GO:0043197**
C=165;O=7;E=0.52;R=13.45;rawP=9.09e-07;adjP=1.83e-05	
Atp1a2	ATPase, Na+/K+ transporting, alpha 2 polypeptide
Fmr1	fragile X mental retardation syndrome 1
Dlgap3	discs, large (Drosophila) homolog-associated protein 3
Frmpd4	FERM and PDZ domain containing 4
Itpr1	inositol 1,4,5-trisphosphate receptor 1
Sipa1l1	signal-induced proliferation-associated 1 like 1
Slc8a1	solute carrier family 8 (sodium/calcium exchanger), member 1

**cellular comp**	**SYNAPSE GO:0045202**
C=555;O=7;E=1.75;R=4.00;rawP=0.0018;adjP=0.0065	
Atp1a2	ATPase, Na+/K+ transporting, alpha 2 polypeptide
Tanc1	tetratricopeptide repeat, ankyrin repeat and coiled-coil containing 1
Fmr1	fragile X mental retardation syndrome 1
Dlgap3	discs, large (Drosophila) homolog-associated protein 3
Itpr1	inositol 1,4,5-trisphosphate receptor 1
Sipa1l1	signal-induced proliferation-associated 1 like 1
Doc2b	double C2, beta

**KEGG pathway**	**CALCIUM SIGNALING PATHWAY 4020**
C=178;O=3;E=0.24;R=12.50;rawP=0.0018;adjP=0.0025	
Itpr1	inositol 1,4,5-trisphosphate receptor 1
Slc8a1	solute carrier family 8 (sodium/calcium exchanger), member 1
Cacna1g	calcium channel, voltage-dependent, T type, alpha 1G subunit

**KEGG pathway**	**REGULATION OF ACTIN CYTOSKELETON 4810**
C=216;O=3;E=0.29;R=10.30;rawP=0.0032;adjP=0.0032	
Arhgef6	Rac/Cdc42 guanine nucleotide exchange factor (GEF) 6
Fgf11	fibroblast growth factor 11
Grlf1	glucocorticoid receptor DNA binding factor 1

**KEGG pathway**	**FOCAL ADHESION 4510**
C=200;O=3;E=0.27;R=11.13;rawP=0.0026;adjP=0.0030	
Col1a1	collagen, type I, alpha 1
Ccnd1	cyclin D1
Grlf1	glucocorticoid receptor DNA binding factor 1

**cellular comp**	**EXTRACELLULAR REGION GO:0005576**
C=1831;O=16;E=5.77;R=2.77;rawP=0.0002;adjP=0.0013	
Masp1	mannan-binding lectin serine peptidase 1
Col17a1	collagen, type XVII, alpha 1
Klk8	kallikrein related-peptidase 8
Inhbb	inhibin beta-B
Itih3	inter-alpha trypsin inhibitor, heavy chain 3
Sema3e	sema domain, immunoglobulin domain (Ig), short basic domain, secreted, (semaphorin) 3E
Npc2	Niemann Pick type C2
Sfrp5	secreted frizzled-related sequence protein 5
Igsf21	immunoglobulin superfamily, member 21
Serpina3n	serine (or cysteine) peptidase inhibitor, clade A, member 3N
Lsr	lipolysis stimulated lipoprotein receptor
Cml3	camello-like 3
Igfbp2	insulin-like growth factor binding protein 2
Col1a1	collagen, type I, alpha 1
Spon1	spondin 1, (f-spondin) extracellular matrix protein
Efemp2	epidermal growth factor-containing fibulin-like extracellular matrix protein 2

**cellular comp**	**CELL JUNCTION GO:0030054**
C=731;O=9;E=2.31;R=3.90;rawP=0.0005;adjP=0.0025	
Col17a1	collagen, type XVII, alpha 1
Tanc1	tetratricopeptide repeat, ankyrin repeat and coiled-coil containing 1
Dlgap3	discs, large (Drosophila) homolog-associated protein 3
Sipa1l1	signal-induced proliferation-associated 1 like 1
Slc8a1	solute carrier family 8 (sodium/calcium exchanger), member 1
Cdc42bpa	CDC42 binding protein kinase alpha
Cdh3	cadherin 3
Esam	endothelial cell-specific adhesion molecule
Ccnd1	cyclin D1

**KEGG pathway**	**ARACHIDONIC ACID METABOLISM 590**
C=90;O=3;E=0.12;R=24.73;rawP=0.0003;adjP=0.0007	
Alox12b	arachidonate 12-lipoxygenase, 12R type
Cyp4f15	cytochrome P450, family 4, subfamily f, polypeptide 15
Ltc4s	leukotriene C4 synthase

[1] E. Sanz, L. Yang, T. Su, D.R. Morris, G.S. McKnight, and P.S. Amieux, Cell-type-specific isolation of ribosome-associated mRNA from complex tissues. Proc Natl Acad Sci U S A 106 (2009) 13939-44.

[2] E.J. Mientjes, I. Nieuwenhuizen, L. Kirkpatrick, T. Zu, M. Hoogeveen-Westerveld, L. Severijnen, M. Rife, R. Willemsen, D.L. Nelson, and B.A. Oostra, The generation of a conditional Fmr1 knock out mouse model to study Fmrp function in vivo. Neurobiol Dis 21 (2006) 549-55.

[3] L. Madisen, T.A. Zwingman, S.M. Sunkin, S.W. Oh, H.A. Zariwala, H. Gu, L.L. Ng, R.D. Palmiter, M.J. Hawrylycz, A.R. Jones, E.S. Lein, and H. Zeng, A robust and high-throughput Cre reporting and characterization system for the whole mouse brain. Nat Neurosci 13 (2010) 133-40.

[4] H. Luuk, S. Koks, M. Plaas, J. Hannibal, J.F. Rehfeld, and E. Vasar, Distribution of Wfs1 protein in the central nervous system of the mouse and its relation to clinical symptoms of the Wolfram syndrome. The Journal of comparative neurology 509 (2008) 642-60.
